# Development of [^18^F]ICMT-11 for Imaging Caspase-3/7 Activity during Therapy-Induced Apoptosis

**DOI:** 10.3390/cancers12082191

**Published:** 2020-08-06

**Authors:** Segundo Francisco García-Argüello, Beatriz Lopez-Lorenzo, Bart Cornelissen, Graham Smith

**Affiliations:** 1 Centro de Investigaciones Médico-Sanitarias, Fundación General Universidad de Málaga, 29010 Málaga, Spain; SFGARCIA@FGUMA.ES; 2 Grupo de Arteriosclerosis, Prevención Cardiovascular y Metabolismo, Instituto de Investigación Biomédica de Málaga (IBIMA), 29010 Málaga, Spain; 3 Biomedicina, Investigación Traslacional y Nuevas Tecnologías en Salud, Universidad de Málaga, 29016 Málaga, Spain; b.lopez.lorenzo@gmail.com; 4 BIONAND-Centro Andaluz de Nanomedicina y Biotecnología (Junta de Andalucía—Universidad de Málaga), 29590 Málaga, Spain; 5 Department of Oncology, CRUK/MRC Oxford Institute for Radiation Oncology, University of Oxford, Old Road Campus Research Building, Off Roosevelt Drive, Oxford OX3 7LJ, UK; bart.cornelissen@oncology.ox.ac.uk

**Keywords:** positron emission tomography, apoptosis, caspase-3, [^18^F]ICMT-11, pyrimidoindolone, Michael acceptor

## Abstract

Insufficient apoptosis is a recognised hallmark of cancer. A strategy to quantitatively measure apoptosis in vivo would be of immense value in both drug discovery and routine patient management. The first irreversible step in the apoptosis cascade is activation of the “executioner” caspase-3 enzyme to commence cleavage of key structural proteins. One strategy to measure caspase-3 activity is Positron Emission Tomography using isatin-5-sulfonamide radiotracers. One such radiotracer is [^18^F]ICMT-11, which has progressed to clinical application. This review summarises the design and development process for [^18^F]ICMT-11, suggesting potential avenues for further innovation.

## 1. Introduction

Evasion of apoptosis is defined as a hallmark of cancer and strategies to image this process in relevant cancer models have occupied scientists for over a decade [1]. The reason for this interest is palpable; most chemotherapeutic treatments act to induce apoptosis and a strategy to quantitatively image this process would facilitate clinical evaluation of patient response [2]. In addition, the strategy could be extended to the evaluation of new therapeutic interventions with an apoptotic endpoint in both pre-clinical and clinical situations [3]. In contrast to the oncology setting, in cardiology and neurology excessive apoptosis is a pathological condition; the ability to quantify therapeutic intervention to minimise apoptotic activity is therefore of potential interest in these disease areas [4].

Apoptosis is triggered by the death receptors extrinsic pathway or the mitochondria-directed intrinsic pathway (induction phase) [5]. Both pathways finally activate caspase-3 and caspase-7, which are effector cysteine-dependent aspartate-directed proteases [6]. Caspases are expressed in normal cells as inactive zymogens but these enzymes are activated in response to specific apoptotic signals (caspase cascade). Caspase-3 is the main executer of apoptosis and cleaves substrates that participate in DNA repair such as poly [ADP-ribose] polymerase 1, as well as a large number of central structural proteins. Multiple divergent strategies have been investigated for apoptosis imaging including imaging agents targeting externalisation of phosphatidylserine and phosphatidylethanolamine, collapse of mitochondrial membrane potential or DNA fragmentation [7,8]. Others have investigated the potential of caspase-3/7 selective isatin-5-sulfonamides for imaging apoptosis [9]. Caspase-3 activation is often considered as the first irreversible step in the apoptotic cascade. Isatin-5-sulfonamides bind with high selectivity and nanomolar affinity for activated caspase-3/7 and form a thiohemiketal between the C-3 carbonyl on the indole ring and a free cysteine thiol at the enzyme active site, thus reflecting activated enzyme expression [10]. 

**Figure 1:**
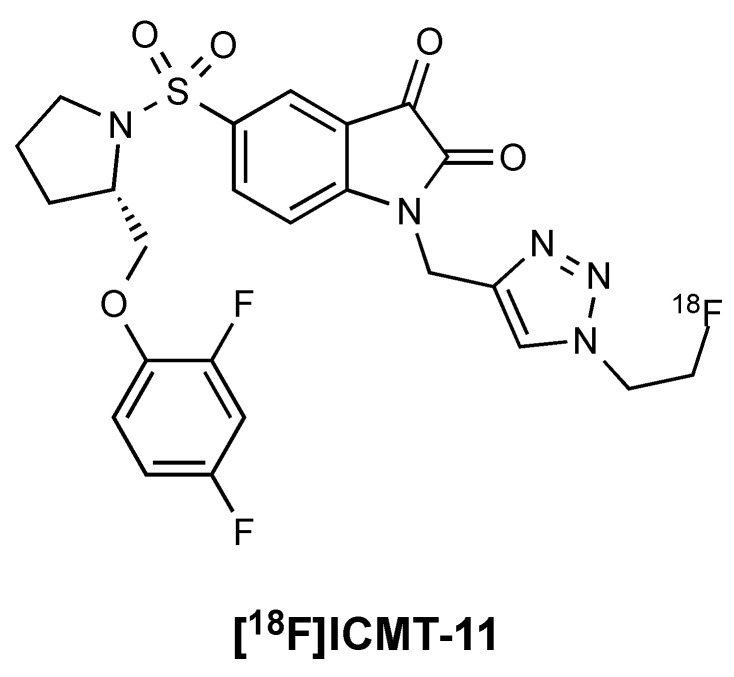
Structure of the caspase-3 PET radiotracer [^18^F]ICMT-11.

Researchers at Imperial College identified a lead candidate radiotracer, [^18^F]ICMT-11, with sub-nanomolar affinity for caspase-3, facile route to radiosynthesis, and tolerable metabolic stability (Figure 1) [11]. [^18^F]ICMT-11 was subsequently evaluated in clinically relevant tumour models, examining the time course for imaging drug-induced apoptotic response and explored methods for voxel-based analysis of Positron Emission Tomography (PET) imaging data [12]. A good manufacturing practice (GMP)-compatible radiosynthetic strategy was eventually designed using the GE FASTlab platform, and [^18^F]ICMT-11 has now progressed into clinical trial [13]. 

## 2. Caspase-3/7 PET Imaging

**Figure 2:**
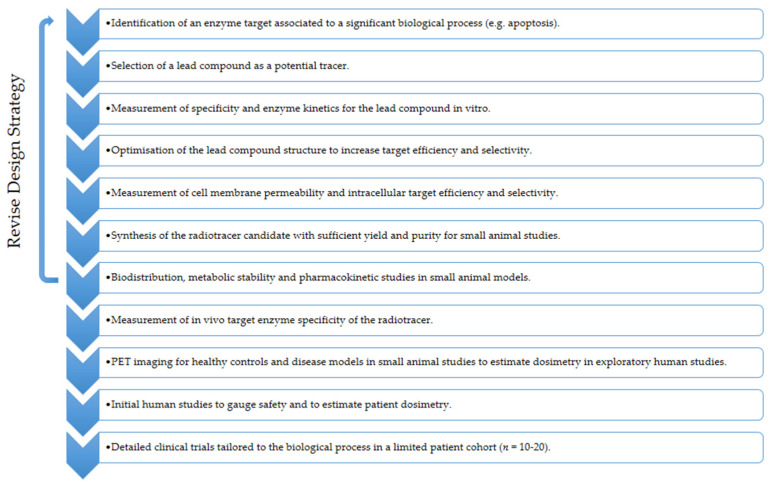
Development process for successful enzyme radiotracers.

PET radiotracers to image enzyme activity can be classified into three groups: substrates, reversible inhibitors and irreversible inhibitors. The design and development of novel PET radiotracers is arduous and shares certain analogies with the drug development process (Figure 2) [14]. Potential radiopharmaceuticals for imaging enzymatic targets should display high affinity and specificity toward the biological target. Additionally, suitable radiolabelling procedures that allow the production of the radiotracer in sufficient activity and purity for in vitro and in vivo preclinical evaluation must be implemented. In the case of favourable in vivo preclinical findings (high metabolic stability, specific tumour retention, toxicology and dosimetry), the final hurdle in the development of novel PET radiotracers lies in the challenge to obtain the approval from relevant regulatory bodies for clinical assessment and validation. 

## 3. Chemical Design and Radiolabelling of [^18^F]ICMT-11

**Figure 3:**
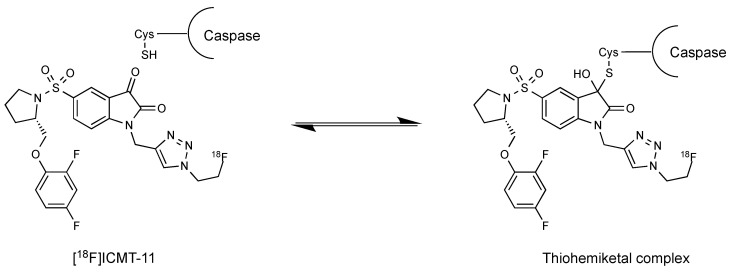
Mechanism of isatin reversible binding to caspases through formation of a thiohemiketal complex.

Isatins are nonpeptidic compounds that reversibly alkylate the thiol group in the active site of caspases-3/7. Lee et al. inserted the sulfonamide functionality at the 5-position of the isatin ring to maintain the appropriate electrophilicity for the warhead carbonyl and enable further molecular diversification into the S2–S4 enzyme pockets [15]. Their mechanism of inhibition is believed to involve the formation of a tetrahedral thiohemiketal intermediate after reaction between the electrophilic C-3 carbonyl group of the isatin moiety and the catalytic cysteine residue (Figure 3). 

Isatin sulfonamide-based PET radiotracers targeting active caspase-3 have been extensively investigated in preclinical studies; a selection is summarised in Table 1 [11,16,17,18,19,20,21,22,23,24,25,26,27,28]. Although there is one example of a boron-dipyrromethene (abbreviated as BODIPY) attachment, most derivations at the N1 position feature 5/6-membered triazole and phenyl rings. At the 5-isatin position, early studies by Lee et al. [15] demonstrated the importance of the pyrrolidine ring for optimal binding in the S2 enzyme pocket and the *S* configuration of the 2′-substitution for binding in the S3 domain. Docking studies suggest that exploitation of hydrophobic interactions in the S2 pocket is crucial to the selectivity of isatin-5-sulfonamides for caspase-3/7. There is a wide scope for substitution at the pyrrolidine-2-ether position with both alkyl and aromatic groups investigated; many of the studies reported to date have used this position to tailor the pharmacokinetic properties of the molecule. The original study by Lee et al. suggested that further derivation in the S3 and S4 pockets could lead to selective differentiation between caspase-3 and caspase-7, but to date, this has not been demonstrated. 

A further avenue of investigation that has received some interest is derivation of the key isatin C-3 carbonyl position essential for reversible binding to the caspase-3 thiol. The Mach group condensed isatin-5-sulfonamides with malonitrile to give a new class of compounds termed “Isatin Michael Acceptors” capable of irreversible binding to thiol residues such as those found at the caspase active site [28]. This class of compounds have lower log P than isatin analogues, which may facilitate the permeation of the radiotracer into the cell and also facilitate non-target clearance, improving in vivo imaging profile as a result. These agents retained high affinity for caspase-3/7, but also some enhanced affinity for caspase-6 which may confound imaging data [29]. 

Inspired by a study from Wyeth, Udemba et al. investigated the pyrimidoindolones as an alternative caspase-3/7 binding scaffold [25]. In contrast to isatin-5-sulfonamides, key advantages of pyrimidoindolones are that this class of inhibitor possesses equal affinity across both halves of a caspase-3 dimer and reduced electrophilicity in the C3 carbonyl warhead, which might decrease non-specific reactions with other thiol-containing residues. The scope for further investigation of this class of inhibitor includes the possibility of additional modulation of the electrophilicity of the “warhead” carbonyl responsible for caspase-binding with the aim of optimising enzyme dissociation kinetics to improve imaging profile.

**Figure 4:**
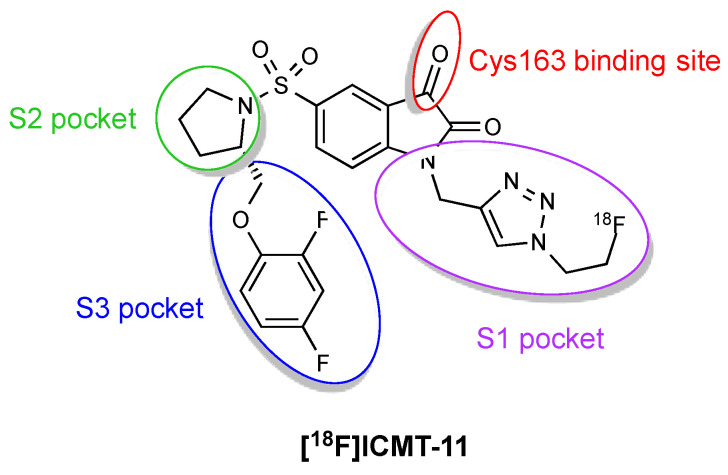
Pharmacophore model for [^18^F]ICMT-11 binding to caspase-3/7.

One of the main problems with the isatin sulfonamide inhibitors is their tendency to biological metabolic degradation, primarily cyctochrome P450-mediated oxidation of the left side ether moiety. To overcome this handicap, a novel series of compounds derived from the isatin 5-sulfonamide scaffold was synthesised and screened for caspase-3 inhibitory affinity [11]. Modifications were made to the left side ether moiety to introduce fluorine substitution on the phenyl ether or use electron-deficient aromatics (e.g., pyridine) more resistant to oxidative metabolism and at the N-1 position to insert the metabolically sTable 1,2,3-triazole (Figure 4). ICMT-11 emerged as the most potent inhibitor, showing improved metabolic profile, reduced lipophilicity (logP = 1.61) compared to common isatin sulfonamide inhibitors (logP ~2.5–3.5) and nanomolar affinity for activated caspase-3 and caspase-7 (IC_50_ = 0.5 nM and 2.5 nM, respectively). ICMT-11 binds to caspase-3/7 with a 10,000-fold selectivity over other caspases, due to binding of the pyrrolidine ring to the S2 hydrophobic pocket of caspase-3/7. 

**Scheme 1:**
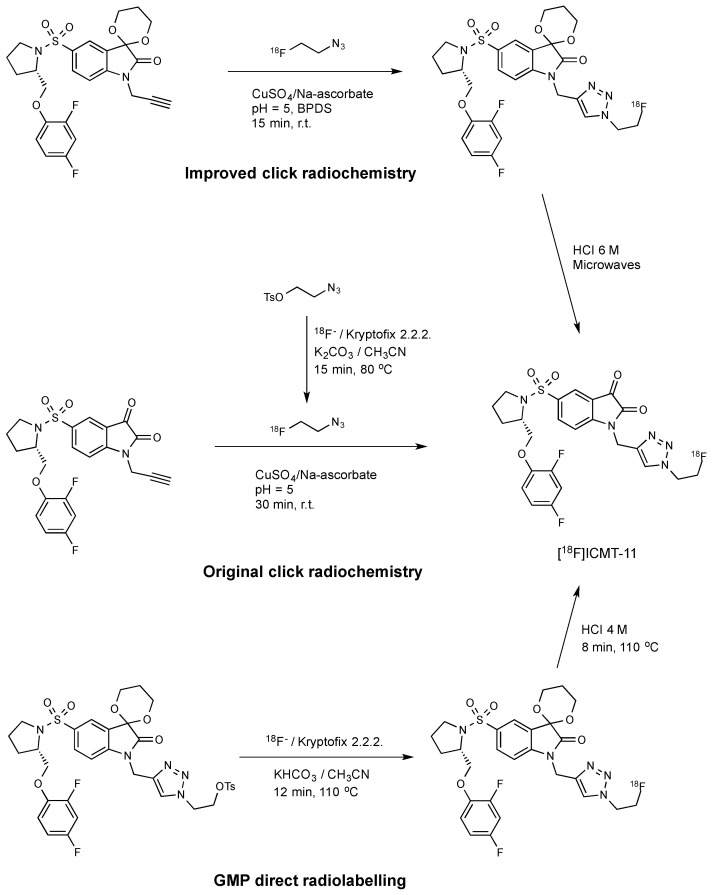
Radiolabelling routes for the synthesis of [^18^F]ICMT-11 [11,30,31].

The original radiosynthesis of [^18^F]ICMT-11 was carried out via a copper catalysed “click chemistry” approach that involved the reaction between an isatin sulfonamide alkyne precursor and 2-[^18^F]fluoroethylazide (Scheme 1) [11]. This synthesis yielded modest molar activity (~1 GBq/µmol) and a non-radioactive isatin analogue which could not be removed from the fluorine-18 radiotracer. This material might potentially compete in the caspase-3 binding; therefore, the initial protocol was subsequently optimised by introducing bathophenanthroline disulfonic acid disodium ligand (BPDS) as an additive to stabilise the Cu(I) catalyst together with acetal protection of the reactive isatin C-3 carbonyl [30]. These modifications minimised stable by-product formation and reduced the amount of alkyne precursor required for the radiolabelling; the overall effect was to raise the molar activity to ~10 GBq/µmol.

Robust and reliable automated procedures for the synthesis of [^18^F]ICMT-11 are essential for the validation and clinical translation of this radiotracer. The radiosynthesis of [^18^F]ICMT-11 under Good Manufacturing Production (GMP) conditions was achieved by the SN_2_ displacement of a protected tosylate precursor with [^18^F]fluoride ion followed by acid deprotection of the acetal protecting group [31]. This route provided [^18^F]ICMT-11 with an improved purity profile and suitable for clinical use. A non-decay corrected radiochemical yield of 3.0 ± 2.6% and a molar activity of 685 ± 237 GBq/μmol were obtained within 90 min. Moreover, radiochemical purity was higher than 95% after several hours, which allows centralised manufacture and distribution to multiple sites for preclinical and clinical evaluation.

## 4. Preclinical Characterisation of [^18^F]ICMT-11 as a Caspase-3-Specific Radiotracer

Initial testing of [^18^F]ICMT-11 in cell-based assays and stability in mice were described in 2008 [11]. Radiotracer uptake was evaluated in radiation-induced fibrosarcoma (RIF-1) untreated cells and under cisplatin-induced apoptosis. A 1.5-fold increase in uptake was observed in treated compared to untreated cells. Subsequent in vivo studies were performed in control and cisplatin-treated RIF-1 tumour-bearing mice, and in this case, the accumulation of [^18^F]ICMT-11 in treated tumours was 2.9-fold higher compared to control animals (Table 2). Biodistribution studies with [^18^F]ICMT-11 indicated that renal and hepatic elimination routes were dominant as suggested by the high uptake detected in kidneys, small intestine, urine and liver after injection. Physiological apoptosis of enterocytes at the villus tips and of macrophages within the intestinal lamina propria may also contribute to the high uptake observed in the small intestine [32]. On the other hand, the radiopharmaceutical was rapidly cleared from the heart and untreated tumours, and defluorination was not observed in vivo. The reasonable stability of [^18^F]ICMT-11 to in vivo metabolic degradation may be related to the presence of the 2′-fluoroethyl-1,2-3-triazole and 2,4-difluorophenyl ether moieties in this compound, since it is known that the introduction of fluorine in aromatic groups reduces considerably metabolic attack by cytochromes P450 [33,34].

**Figure 5:**
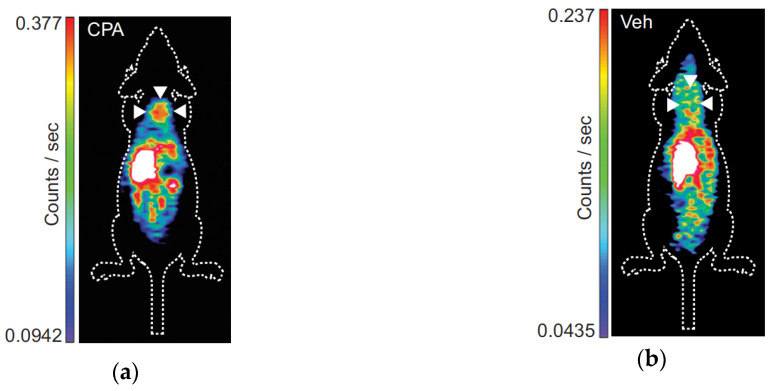
[^18^F]ICMT-11 PET images of 38C13 xenograft-bearing mice: (**a**) Treated with CPA; (**b**) Treated with vehicle.

Nguyen et al. further characterised the [^18^F]ICMT-11 uptake in different cell lines treated with anticancer agents [35]. Validation of radiotracer specificity for activated caspase-3 was demonstrated by the invariable uptake in caspase-3-deficient MCF-7 human breast cancer cells treated with the alkylating agent 4-hydroperoxycyclophosphamide (4-HC) compared to untreated cells. Otherwise, human pulmonary carcinoma LNM35 cells treated with etoposide or cisplatin showed a 2-fold increase in [^18^F]ICMT-11 binding compared to controls. A 2-fold radiotracer binding increase compared to controls was also observed in 38C13 murine lymphoma cells that were treated with 4-HC and cell uptake was not related to increased cell permeability in apoptotic cells. This increased radiotracer signal was corroborated by in vivo PET imaging of 38C13 xenograft-bearing mice treated with cyclophosphamide (CPA) or vehicle (Figure 5). A posterior study showed that the optimal time-point for detection of caspase-3 activation with [^18^F]ICMT-11 in this lymphoma model was after 24 h of CPA treatment [12].

[^18^F]ICMT-11 has also been evaluated in murine lymphoma EL4 tumour-bearing mice 24 h after treatment with a combination of etoposide/cyclophosphamide [30]. PET imaging revealed a 1.4-fold increased uptake in the drug-treated animals and remarkable heterogeneous distribution of apoptosis within the tumours. Hence, voxel-wise analysis of the radiotracer spatial distribution in tumours was proposed to be more relevant for the investigation of caspase-3 activation.

Birinapant is a second mitochondria-derived activator of caspases (SMAC) mimetic that generates caspase activation and apoptosis [40]. The effect of birinapant on caspase-3 activation was evaluated with [^18^F]ICMT-11 in mice bearing HCT116 colon carcinoma or MDA-MB-231 breast adenocarcinoma tumours [12]. In both cases, a 1.5-fold increase in tumour uptake was detected at 6 hours after treatment compared to baseline. However, the radioactive signal returned to baseline levels at later times post-treatment (24–48 h); the authors attribute this to the transitory nature of caspase-3 activation (owing to enzyme self-degradation) although no ex vivo analysis of caspase-3 activity was carried out.

[^18^F]ICMT-11 has been used to analyse the effects of single or repeated doses of the antiepidermal growth factor receptor antibody cetuximab in mice bearing cetuximab-sensitive H1975 tumours (non–small cell lung cancer) or cetuximab-insensitive HCT116 tumours [36]. Unsurprisingly, no detectable change in tumour uptake was observed in the HCT116 cohort after treatment due to the known minimal sensitivity to cetuximab treatment of this cancer model [41]. Moreover, there was a significant increase in tumour [^18^F]ICMT-11 intensity in H1975 tumour-bearing mice after repeated dosing with cetuximab as monotherapy or combination therapy with gemcitabine. Although changes in radiotracer uptake were small and heterogenous, they correlated with the ex vivo staining of cleaved caspase-3. 

Carboplatin-induced apoptosis in PC9 and A549 human non-small cell lung cancer models has been also assessed by [^18^F]ICMT-11 [37]. Radiotracer uptake in PC9 cells was dose-dependent and increased up to 14-fold compared to vehicle treated cells. Time course evaluation of carboplatin treatment in PC9 cells revealed that the increase in [^18^F]ICMT-11 accumulation paralleled the temporal increase in caspase-3 activity up to 72 h post-treatment. Owing to the dominant presence of pre-therapy necrosis, only a 1.5-fold average increase in the number of voxels with high intensity uptake was detected in vivo in treated PC9 xenograft-bearing mice compared to vehicle. In contrast, the necrotic pathway was the predominant death mechanism in A549 cells and in A549 tumour-bearing mice, thus, no changes in radiotracer uptake were found following carboplatin therapy. 

The mouse B16ovaRevC3 melanoma cell line is generated by transfection of the ovalbumin-expressing mouse B16 melanoma cell line (B16ova) with a tetracycline-regulated reverse transcriptional transactivator that activates caspase-3 [42]. Induction of this death-switch by doxycycline results in rapid and synchronous apoptosis both in vitro and in vivo in syngeneic murine melanoma lines. The uptakes of [^18^F]ICMT-11 and the apoptosis radiotracer [^18^F]ML-10 were evaluated in B16ova and B16ovaRevC3 cells with and without doxycycline [38]. Although the mechanism of action for [^18^F]ML-10 remains unclear, it is postulated to function through attraction of the anionic head group to positively charged phosphatidylserine residues on the surface of apoptotic cells followed by “anchoring” of the lipophilic tail in the permeable membrane of the dying cell. A direct comparison between [^18^F]ML-10 and [^18^F]ICMT-11 would be advantageous, but as of yet, this has not been done. A 4.5-fold increase in the cellular uptake of [^18^F]ICMT-11 following death-switch induction in B16ovaRevC3 cells was observed. Otherwise, just a 1.5-fold increase in the uptake of [^18^F]ML-10 in B16ovaRevC3 cells after doxycycline treatment was detected, confirming the superiority of [^18^F]ICMT-11 in this experimental model. In contrast, doxycycline treatment made no difference in the uptake of any of the radiotracers in B16ova cells. Similar to the in vitro results, PET imaging with [^18^F]ICMT-11 showed higher tumour uptake than [^18^F]ML-10 in mice B16ovaRevC3 xenografts after treatment. However, only a 2.2-fold increase in [^18^F]ICMT-11 tumour accumulation was detected in vivo after doxycycline administration, which was significantly lower compared to the in vitro results. The reasons for this discrepancy are unclear and might be a result of several factors, such as induction of changes within the tumour microenvironment, quick progression towards secondary necrosis, rapid clearance of apoptotic cells by host phagocytes, or other confounding factors occurring in vivo.

Mycobacterium tuberculosis is known to manipulate the host cell to induce necrosis by preventing apoptosis [43,44]. Therefore, modulation of the immune response by promoting pro-apoptosis pathways may be applied as a potential therapy in tuberculosis [45]. [^18^F]ICMT-11 has been recently used to evaluate the pro-apoptotic reaction in cells and mice infected with Mycobacterium tuberculosis and treated with cisplatin [39]. Infected cells and animals had lower radiotracer uptake compared to uninfected controls, possibly due to the mycobacterial induction of anti-apoptotic proteins. Nonetheless, the pro-apoptotic response induced by cisplatin in tuberculosis-infected mice generated a 1.4-fold increase in the [^18^F]ICMT-11 signal. These results may be helpful for the evaluation of relevant pro-apoptotic agents in the treatment of pulmonary tuberculosis.

The preclinical studies summarised above demonstrate a typical increase in [^18^F]ICMT-11 of 1.5–2.5-fold over background in apoptotic tumours in vivo. This level of uptake is sufficient to detect apoptosis under optimised conditions but not to quantitatively assess a spectrum of apoptotic response. A recent analysis by Gammon et al. suggests that the low uptake of [^18^F]ICMT-11 is a fundamental limitation of the caspase-3 ligand strategy and is unlikely to be solved by further molecular diversification [46]. In the relevant study the predictive power of apoptosis imaging agents was postulated as being a function of both biochemical suitability but also underlying temporal compatibility with apoptotic induction. In summary, imaging agents with a short (~hours) reporting window were more susceptible to timing variability in therapy-induced tumour apoptosis, particularly given the typical small differences in apoptotic activity between treated and untreated tumours (~2.9-fold).

## 5. Clinical Translation of the Radiotracer: Human Studies with [^18^F]ICMT-11

The promising mechanistic and biologic profile of [^18^F]ICMT-11 supported its transition into clinical development. Evaluation in healthy human volunteers has showed that the radiotracer is safe and presents a satisfactory radiation dosimetry profile for clinical imaging that is comparable to other common fluorine-18 PET radiotracers such as [^18^F]FDG [13]. After the administration of [^18^F]ICMT-11, radioactivity rapidly distributes to the liver and kidneys, followed by rapid elimination through the kidneys and the hepatobiliary system. The organs receiving the highest absorbed dose are the gallbladder wall, small intestine, upper large intestinal wall, urinary bladder wall and liver. The high retention of the tracer shown in the liver and intestines due to slow washout into the gastrointestinal tract and hepatic-based metabolism with gut clearance might limit interpretation of [^18^F]ICMT-11 PET images of the abdominal region. However, a physiologic activity reduction in the liver at earlier time points and increased activity in the bowel was observed in subjects that had a meal prior to radiotracer injection. Therefore, incorporation of a standard pre-scan meal or a promotility agent in the scanning protocol may be helpful to improve abdominal imaging with this radiotracer.

**Figure 6:**
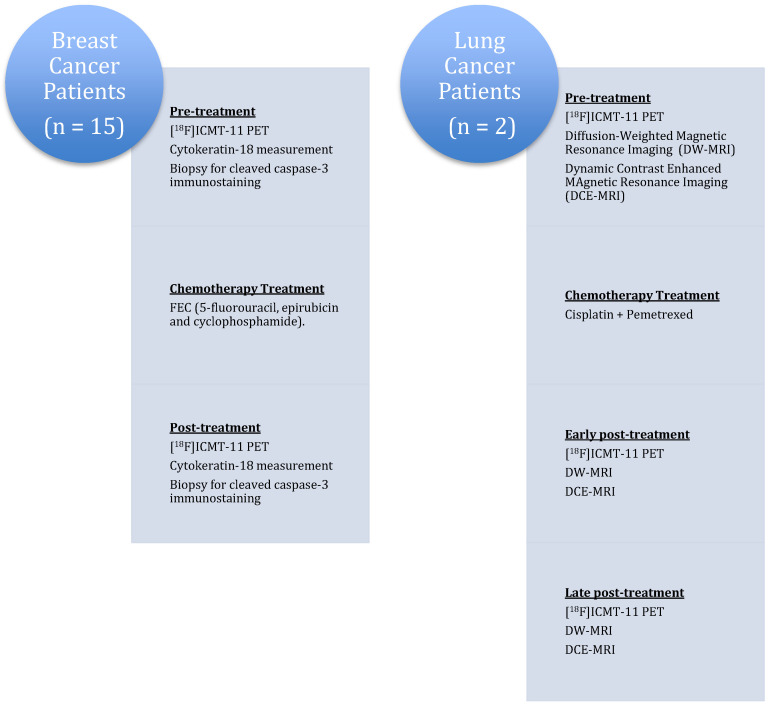
Clinical study design to evaluate the effect of chemotherapy on [^18^F]ICMT-11 uptake.

The results of the first clinical study investigating tumour apoptosis with [^18^F]ICMT-11 have been reported recently in lung cancer patients receiving chemotherapy and in women with advanced breast cancer (invasive ductal carcinoma) before and after receiving the first cycle of neoadjuvant chemotherapy (Figure 6) [47]. Due to the heterogeneous response of the tumours after treatment, the analysis of apoptosis in PET imaging was achieved using a PET-based voxel intensity sorting (PVIS) approach [12]. Higher voxel intensity in treated tumours compared to baseline was assigned PVIS apoptosis-dominant signature (PADS), while lower voxel intensity was assigned PVIS necrosis-dominant signature (PNDS).

The small group of lung cancer patients consisted of subjects diagnosed with non-small cell lung cancer undergoing platinum-based chemotherapy treatment (pemetrexed and cisplatin). The longitudinal effect of first-line chemotherapy in apoptosis was measured with [^18^F]ICMT-11, diffusion-weighted and dynamic contrast-enhanced magnetic resonance imaging (DW-MRI and DCE-MRI) at each time point. Patients were scanned at 24 h and at 7 days after the first cycle of chemotherapy. [^18^F]ICMT-11 uptake and apparent diffusion coefficient values in tumours extracted from the MRI studies appeared to be congruent in the lung cancer patients.

In the breast cancer cohort, the study assessed if the effect of FEC-T (5-fluorouracil, epirubicin, cyclophosphamide and docetaxel) neoadjuvant treatment on [^18^F]ICMT-11 uptake correlated with blood cytokeratin-18 assessment and biopsy-derived caspase-3/7 tissue expression following chemotherapy. Patients were divided into early (24–48 h) and late (2–14 days) imaging post-chemotherapy. Cytokeratin-18 analysis in blood samples showed that therapy was predominantly not apoptosis in nature. The majority of breast cancer patients in the study showed a dominant necrotic or mixed apoptotic/necrotic signature phenotype, which may explain the lack of a predominant apoptotic signal on PET and histology.

Altogether, patient results showed that only a small proportion of apoptosis was induced by drug treatment and possible reasons may include:

- Timing and transient nature of apoptosis.
- Heterogeneous activation of apoptosis within tumours.
- Distinct cell death mechanisms other than apoptosis that may be involved in treatment response, such as necrosis, mitotic catastrophe, senescence, autophagy, pyroptosis and DNA damage [48].

Voxel-wise analysis showed regional increases of [^18^F]ICMT-11 intensity regions in some tumours, and while patients having this phenotype responded to therapy, it was not an exclusive marker of response. Thus, tumour response could occur in the absence of predominant chemotherapy-induced caspase-3/7 activation measured non-invasively across entire tumour lesions in patients with breast and lung cancer. Complex voxel analysis may be limited to specialist research applications; routine clinical application would require considerable staff training and extensive validation with established apoptotic indicators.

## 6. Conclusions

Quantitative PET imaging of activated caspases-3/7 may become a valuable tool for early monitoring of tumour response to therapy and can be achieved through radiolabelled enzyme inhibitors or substrates. Several caspase inhibitors have been described in the literature, but only [^18^F]ICMT-11 has reached the clinical stage. This isatin sulfonamide displays high affinity and selectivity for caspase-3 and caspase-7. However, assessment of [^18^F]ICMT-11 uptake in abdominal tumours may be challenging and complex voxel-wise analysis of PET imaging data is necessary due to the heterogeneous response of tumours to therapy. In addition to this, the sensitivity of [^18^F]ICMT-11 is limited despite the specific binding of this PET radiotracer to activated caspases. [^18^F]ICMT-11 tumour uptake decreases over time after tracer administration, thus, increased differences between treated and control tumours are not found at late time points. The low absolute target uptake may be due to interference of apoptosis progression after enzyme inhibition or to the saturation of the binding sites. Otherwise, radiolabelled caspase substrates potentially have less susceptibility to saturation and a single enzyme can interact with more than one substrate molecule within the time frame of a PET study, which may result in the amplification of the radioactive signal produced by the active caspase. Various potential substrate candidates have been investigated in preclinical models and intracellular retention of the cleaved radiotracers has been achieved through different strategies, such as intracellular aggregation or the inclusion of cell-penetrating peptides in the molecule [49,50,51,52,53]. Preclinical results have suggested that it is possible to image apoptosis and therapy-induced increases of caspase-3/7 with substrate radiotracers. However, these radiolabelled substrates have not provided substantial signal improvements with respect to caspase inhibitors, perhaps due to slow permeation of the substrates into tissues and cells.

PET radiotracers that image caspase-3/7 activation are certainly valuable in preclinical studies, but it is unclear if this specificity may be of less importance in the clinical setting. Minor changes in tumour uptake were found in the first clinical study with [^18^F]ICMT-11 in breast and lung cancer patients following chemotherapy. Since anti-cancer drugs provoke cell death through different mechanisms, a head-to-head comparison of response detection to chemotherapeutic agents with PET radiotracers targeting different aspects of cell death should be included in future clinical studies [54]. A further implicit difficulty for the treatment response assessment with cell death radiotracers is the limited knowledge of the optimal post-therapy imaging window and the complex protocols that are necessary for patient evaluation. Furthermore, the magnitude of early response to treatments is not always predictive for the long-term outcome.

Although detection of early treatment efficacy remains a challenge in cancer treatment management, the implementation of [^18^F]ICMT-11 PET imaging into clinical routine can be beneficial, at least in initial patient stratification into responders and non-responders. Moreover, evaluation of treatment response with [^18^F]ICMT-11 may constitute a smart alternative to current apoptosis assessment based on tumour biopsies. Therefore, the usefulness of [^18^F]ICMT-11 needs to be further examined in larger cohorts of well-designed prospective studies.

## Figures and Tables

**Table 1:** List of non-peptidic inhibitors of caspase-3/7.

Mode of Action	Structure	Affinity (IC_50_)	Reference
Isatin Inhibitor	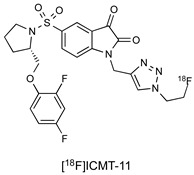	0.5 nM (caspase-3) 2.5 nM (caspase-7)	[11]
Isatin Inhibitor	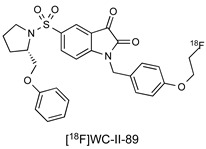	9.7 nM (caspase-3) 23.5 nM (caspase-7)	[16]
Isatin Inhibitor	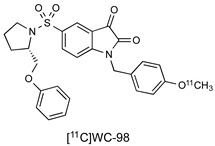	14.5 nM (caspase-3) 22 nM (caspase-7)	[17]
Isatin Inhibitor	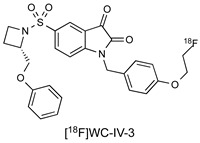	8.6 nM (caspase-3) 26 nM (caspase-7)	[18]
Isatin Inhibitor	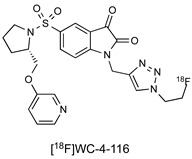	4.5 nM (caspase-3) 3.8 nM (caspase-7)	[19]
Isatin Inhibitor	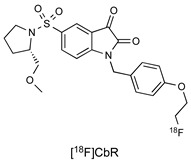	36.4 nM (caspase-3) 93.3 nM (caspase-7)	[20]
Isatin Inhibitor	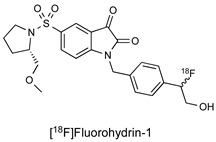	80 nM (caspase-3) 7.6 nM (caspase-7)	[21]
Isatin Inhibitor	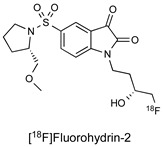	25.9 nM (caspase-3) 296 nM (caspase-7)	[22]
Isatin Inhibitor	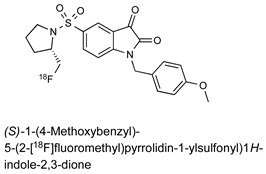	13.7 nM (caspase-3) 9.6 nM (caspase-7)	[23]
Isatin Inhibitor	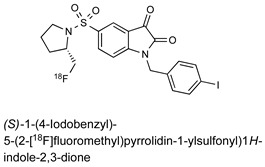	8.1 nM (caspase-3) 5.4 nM (caspase-7)	[24]
Pyrimidoindolone Inhibitor	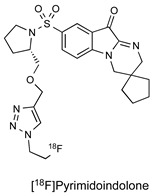	100.4 nM (caspase-3)	[25]
Isatin Inhibitor	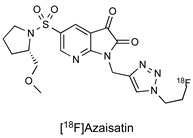	21 nM (caspase-3) 97 nM (caspase-7)	[26]
Isatin Inhibitor	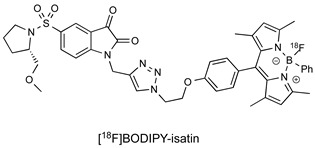	34 nM (caspase-3) 422 nM (caspase-7)	[27]
Isatin Michael Acceptor	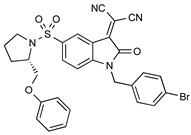	20.1 nM (caspase-3) 92.2 nM (caspase-7)	[28]

**Table 2:** Results of the preclinical evaluation of [^18^F]ICMT-11 in treated tumours.

Preclinical Evaluation	Disease Model	Treatment	Imaging Time after Treatment	Uptake Outcome	Reference
In vitro/In vivo	RIF-1 murine fibrosarcoma	Etoposide or cisplatin	24 h	1.5–2.9-fold increase	[11]
In vitro	LNM35 human pulmonary carcinoma	Etoposide or cisplatin	24 h	2-fold increase	[35]
In vitro	MCF-7 human breast cancer (caspase-3 deficient)	4-HC ^1^	24 h	No change	[35]
In vitro/In vivo	38C13 murine lymphoma	Cyclophosphamide or 4-HC	24–48 h	1.5-2-fold increase	[12,35]
In vivo	EL4 murine lymphoma	Etoposide/cyclophosphamide	24 h	1.4-fold increase	[30]
In vivo	MDA-MB-231 breast adenocarcinoma	Birinapant	24–48 h	1.5-fold increase	[12]
In vivo	HCT116 colon carcinoma	Birinapant	24–48 h	1.5-fold increase	[12]
In vivo	HCT116 colon carcinoma	Cetuximab/gemcitabine	72 h	No change	[36]
In vivo	H1975 NSCLC (cetuximab sensitive)	Cetuximab/gemcitabine	72 h	1.5–1.8-fold increase	[36]
In vitro/In vivo	PC9 NSCLC (carboplatin sensitive)	Carboplatin	24–48 h	1.5–14-fold increase	[37]
In vitro/In vivo	A549 NSCLC (carboplatin insensitive)	Carboplatin	24–48 h	No change	[37]
In vitro/In vivo	B16ovaRevC3 murine melanoma	Doxycycline	24 h	2.2–4.5-fold increase	[38]
In vitro/In vivo	B16ova murine melanoma	Doxycycline	24 h	No change	[38]
In vitro/In vivo	Mycobacterium tuberculosis	Cisplatin	6 h	1.4-fold increase	[39]

^1^ 4-HC: 4-Hydroperoxycyclophosphamide.
